# Opposite effects on facial morphology due to gene dosage sensitivity

**DOI:** 10.1007/s00439-014-1455-z

**Published:** 2014-06-03

**Authors:** Peter Hammond, Shane McKee, Michael Suttie, Judith Allanson, Jan-Maarten Cobben, Saskia M. Maas, Oliver Quarrell, Ann C. M. Smith, Suzanne Lewis, May Tassabehji, Sanjay Sisodiya, Teresa Mattina, Raoul Hennekam

**Affiliations:** 1 Molecular Medicine Unit, UCL Institute of Child Health, 30 Guilford St, London, WC1N 1EH UK; 2 Belfast City Hospital Trust, Belfast, UK; 3 Children’s Hospital of Eastern Ontario, Ottawa, Canada; 4 Department of Pediatrics, Academic Medical Center, University of Amsterdam, Amsterdam, Netherlands; 5 Department of Clinical Genetics, Sheffield Children’s Hospital, Sheffield, UK; 6 National Human Genome Research Institute, National Institutes of Health, Bethesda, USA; 7 Medical Genetics, University of British Columbia, Vancouver, Canada; 8 Manchester Centre for Genomic Medicine, Institute of Human Development, Faculty of Medical and Human Sciences, University of Manchester, Manchester, UK; 9 Central Manchester University Hospitals NHS Foundation Trust, Manchester, UK; 10 Department of Clinical and Experimental Epilepsy, UCL Institute of Neurology, London, UK; 11 Medical Genetics, University of Catania, Catania, Italy

## Abstract

Sequencing technology is increasingly demonstrating the impact of genomic copy number variation (CNV) on phenotypes. Opposing variation in growth, head size, cognition and behaviour is known to result from deletions and reciprocal duplications of some genomic regions. We propose normative inversion of face shape, opposing difference from a matched norm, as a basis for investigating the effects of gene dosage on craniofacial development. We use dense surface modelling techniques to match any face (or part of a face) to a facial norm of unaffected individuals of matched age, sex and ethnicity and then we reverse the individual’s face shape differences from the matched norm to produce the normative inversion. We demonstrate for five genomic regions, 4p16.3, 7q11.23, 11p15, 16p13.3 and 17p11.2, that such inversion for individuals with a duplication or (epi)-mutation produces facial forms remarkably similar to those associated with a deletion or opposite (epi-)mutation of the same region, and vice versa. The ability to visualise and quantify face shape effects of gene dosage is of major benefit for determining whether a CNV is the cause of the phenotype of an individual and for predicting reciprocal consequences. It enables face shape to be used as a relatively simple and inexpensive functional analysis of the gene(s) involved.

**Electronic supplementary material:**

The online version of this article (doi:10.1007/s00439-014-1455-z) contains supplementary material, which is available to authorized users.

## Introduction

In recent years, genome-wide screening technologies have helped identify large numbers of genomic structural variants (GSVs). Duplications and deletions, usually indicated as copy number variants (CNVs), are the most prevalent GSVs and have been shown to make an important contribution to development and disease (Valsesia et al. 2013; Weischenfeldt et al. 2013). CNVs have been associated with mirrored or opposing phenotypes at several loci. For example, CNVs of the 7q11.23 Williams-Beuren syndrome (OMIM: #194050) region cause neuro developmental disorders with a multi-faceted phenotype and variable expressivity. Typically, individuals with 7q11.23 micro deletions have specific facial dysmorphism, supravalvular aortic stenosis, hypercalcaemia and a distinctive cognitive profile including heightened sociability and relative strength of language over visuo-spatial processing. In contrast, the 7q11.23 reciprocal duplication results in different facial dysmorphism, low sociability and prominent speech delay (Schubert 2009; Merla et al. 2010). This deletion-duplication opposing nature of a phenotype also occurs for 17p11.2. Individuals with Smith-Magenis syndrome (OMIM: #182290), caused by a deletion in 17p11.2 or point mutation in *RAI* at 17p11.2, exhibit attention-seeking and overt friendliness (Potocki et al. 2000, 2007; Lupski and Stankiewicz 2005). In contrast, the reciprocal duplication causes Potocki-Lupski syndrome where behaviour is characterized by autism spectrum disorders. Opposing over/undergrowth effects result from deletions (Sotos syndrome (OMIM: #117550)) and duplications of *NSD1* at 5q35 (Zhang et al. 2011; Rosenfeld et al. 2013; Žilina et al. 2013; Franco et al. 2010; Dikow et al. 2013). Opposing extreme BMI phenotypes have also been associated with gene dosage at 16p11.2 (Jacquemont et al. 2011). In the two latter regions, there is also opposing microcephaly and macrocephaly. Hypomethylation of imprinting control region 1 at 11p15 and maternal duplication of 11p15 have been described as major (epi) genetic disturbances in Silver-Russell syndrome (OMIM: # 180860) resulting in severe undergrowth. Opposite (epi)-mutations involved in Beckwith-Wiedemann syndrome (OMIM: # 130650) cause overgrowth, suggesting that Silver-Russell and Beckwith-Wiedemann syndromes are genetically and clinically opposite (Eggermann 2009).

Based on these observations, we hypothesised that in opposite CNV pairings, there could be quantitatively opposite facial phenotypes. Facial morphology is determined in part by a large number of genes and enhancers acting in concert, and a decrease in dosage in some genes will lead to abnormal morphology at various parts of the face. An increase in dosage may therefore lead to a related abnormal morphology at the same parts of the face. To test this hypothesis, we developed a transformation, normative inversion, for reversing the differences of an individual’s 3D face shape from a facial norm, the average of an age-sex-ethnicity matched control group.

## Methods

### Study participants

3D facial images were available for 387 Caucasian controls and individuals with Williams-Beuren syndrome, Smith-Magenis syndrome and Wolf-Hirschhorn syndrome (OMIM: # 194190) from previous studies (Hammond et al., 2004, 2005, 2012). Individuals with Rubinstein-Taybi syndrome were recruited during attendance at national family support group meetings in the Netherlands, Norway and UK. Through clinical co-authors, we also recruited individuals specifically for this study with confirmed mutations causing Beckwith-Wiedemann (*n* = 1) and Silver-Russell syndromes (*n* = 17) as well as individuals with associated duplications at 4p16.3 (*n* = 1), 7q11.23 (*n* = 4), 16p13.3 (*n* = 1) and 17p11.2 (*n* = 1). All subjects were of Caucasian origin except for one duplication 7q11.23 case which was of North African (Moroccan) origin.

### Normative inversion of facial morphology

Initially, we constructed a dense surface model (DSM) of the 3D facial images of individuals with an identified syndrome or CNV and the 387 controls (Hammond and Suttie 2012). In such models, we retain sufficient principle component analysis (PCA) modes to cover 99 % of all shape variation. The inclusion of a large number of controls and a high proportion of PCA modes enables accurate synthesis of faces in the model.

The subset of controls of the same ethnicity and gender were ordered by age and a series of running mean faces of 50 contiguously aged individuals were computed. This enabled every face to be matched to a same ethnicity-gender facial norm of closely matched age. Within the multi-dimensional DSM representation, all faces were projected onto a hyperline from the face being inverted based at (1, 0) to the matched norm based at (−1, 0). The line was extended to a point the same distance away at (−3, 0) where the face shape corresponds to an inverted facial form whose shape differences from the norm are exactly opposite to those of the original face. Such a normative inversion can be applied to any face.

A similar notion of facial inversion, termed anti-face, was previously used to investigate psychological aspects of the perception of face shape differences (Blanz et al. 2000). In this earlier study, various mean faces were employed to compute an opposite face shape, sometimes even based on a mixed set of male and female individuals. Therefore, we retain the use of “normative inversion” in order to emphasize the age-sex-ethnicity matched nature of the mean used in our inversion process.

**Fig. 1:**
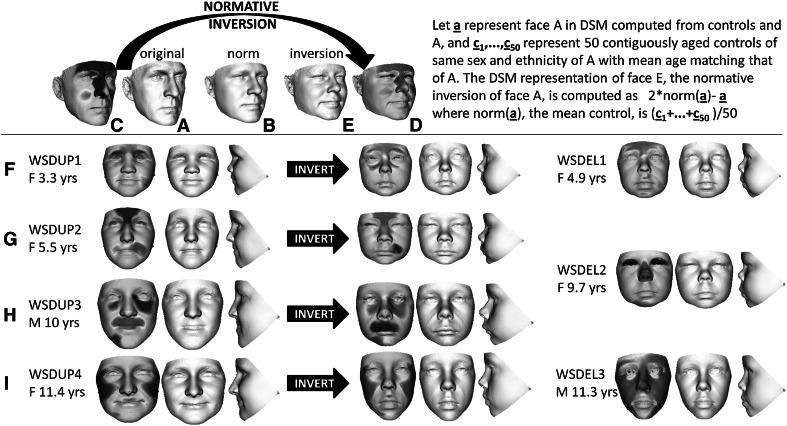
Normative inversion of face shape. **a** The face surface of an adult Caucasian male control. **b** The average face of 50 adult male Caucasians whose mean age matches that of **a**. **c** A heat map of the face signature of **a** normalised against the 50 individuals whose average is **b**. **d** The inverted heat map of **c**. **e**. The face surface whose face signature has heat map **d**. **f** *Left* to *right* a triptych of face signature, portrait and profile of individual 1 with a duplication of 7q11.23; then the normative inversion of the duplication case; and finally an individual with a confirmed deletion of 7q11.23 whose face closely resembles the inversion. **g** *Left* to *right* a triptych of face signature, portrait and profile of individual 2 with a duplication of 7q11.23; then the normative inversion of the duplication case; and finally an individual with a confirmed deletion of 7q11.23 whose face closely resembles the inversion. **h** *Left* to *right* a triptych of face signature, portrait and profile of individual 3 with a duplication of 7q11.23; then the normative inversion of the duplication case. **i** *Left* to *right* a triptych of face signature, portrait and profile of individual 4 with a duplication of 7q11.23; then the normative inversion of the duplication case; and finally an individual with a confirmed deletion of 7q11.23 whose face closely resembles the inversion. Note that in the original and inverted face signatures of rows **f**–**h**, the *red–green–blue* of the heat maps are opposite with *red* and *blue* regions interchanged. The *red–green–blue* spectrum in all images represents regions of contraction-coincidence-expansion relative orthogonal to the face surface of the matched norm with extreme *red-blue* indicating difference beyond 2SD (color figure online)

The face signature of an individual is the term used for its 3D shape difference from its matched norm (Hammond and Suttie 2012). It can be visualised as a heat map reflecting the normalized differences at each of 25,000 + surface points captured by commercial 3D imaging devices. Figure 1a, c show the face surface and signature heat map of one of the authors where the red–green–blue spectrum highlights regional differences (contraction-coincidence-expansion) orthogonal to the face surface compared to the matched norm. The extremes of red and blue reflect normalised differences of ± 2 SDs or more. Figure 1b shows the matched facial norm and Fig. 1d, e the surface and heat map of the normative inversion of A with respect to B. We apply an analogous transformation to all faces of interest.

## Results

**Fig. 2:**
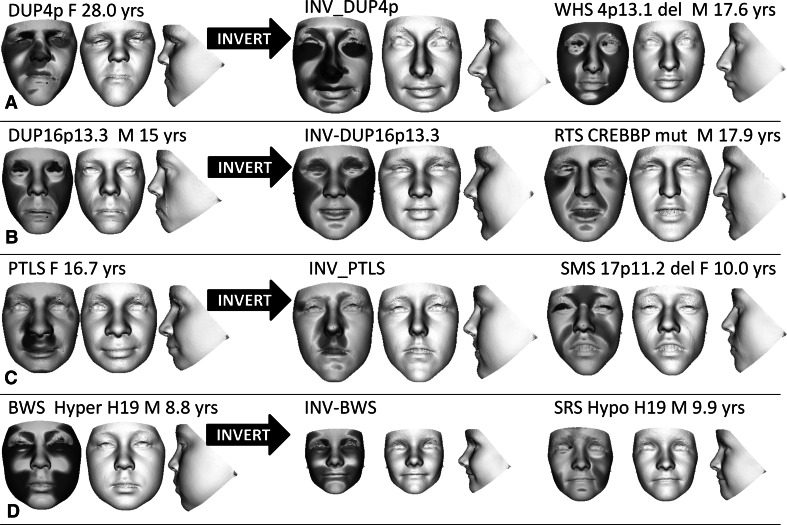
Normative inversion of duplications of 4p16.3, 16p13.3, 17p11.2 and H19 hypermethylation Beckwith-Wiedemann cases. Using the same format as Fig. 1, each row includes a face triptych comprising signature, portrait and profile of a duplication (4p16.3, 16p13.3, 17p11.2) or hypermethylation (Beckwith-Wiedemann syndrome) case; the normative inversion of the original; and, finally a triptych for an individual with a deletion or mutation (Wolf–Hirschhorn syndrome, Rubinstein–Taybi syndrome, Smith–Magenis syndrome) or opposite methylation (Silver–Russell syndrome) whose face shape closely resembles that of the original case. The inversions of the dup 4p16.3 and Beckwith–Wiedemann cases display strong similarity respectively with features of Wolf–Hirschhorn and Silver–Russell syndromes. The inversion of the dup 16p13.3 case displays Rubinstein–Taybi features such as hooded eyes, significant convexity of the zygomatic arch and exposure of the columella. Although somewhat narrower than is usual in Smith–Magenis syndrome, the inversion of the Potocki–Lupski case displays the typical upward curve to the upward lip, the hidden columella and mid-facial flatness

In Fig. 1f–i, we demonstrate the effect of inverting the face shape of three unpublished individuals and one previously published individual with a duplication of 7q11.23 (Malenfant et al. 2012). Clinically, the features of the normative inversions of their faces strongly resemble those of Williams-Beuren syndrome (flat nasal bridge, short upturned nose, long philtrum, full lips, malar flattening, and micro/retrognathia). This similarity is further emphasised by their comparison with three adjacent faces of individuals with a confirmed 7q11.23 Williams-Beuren syndrome deletion. To test normative face inversion further, the procedure was repeated for individuals with duplications of 4p16.3, 16p13.3 and 17p11.1, and with Beckwith-Wiedemann syndrome caused by H19 hypermethylation. Clinical evaluation of the normative inversions of facial features of these cases establishes their similarity to facial characteristics of individuals with Wolf-Hirschhorn (Fig. 2a), Rubinstein-Taybi (Fig. 2b), Smith–Magenis (Fig. 2c) and Silver–Russell syndromes due to H19 hypomethylation (Fig. 2d), respectively.

A convincing objective confirmation of these clinical observations would be to demonstrate the effect of normative inversion along a duplication-deletion axis—for example, classifying each duplication case and its inverse relative to proximity to the mean duplication and mean deletion cases (taking care to omit the test individual from the calculation of the mean duplication). But there are an insufficient number of identified duplication cases available and so a compromise was to measure the effect of inversion of the face of an individual with a duplication along a control-deletion axis. A moment’s reflection confirms that as a result of normative inversion there would be much stronger movement of closest mean classification along a duplication-deletion axis than along a control-deletion axis, as the duplication group mean would be more “repulsing” to the inverted duplication. But until more duplication cases can be identified, the control-deletion axial comparison will have to substitute for the duplication-deletion axial comparison.

**Fig. 3:**
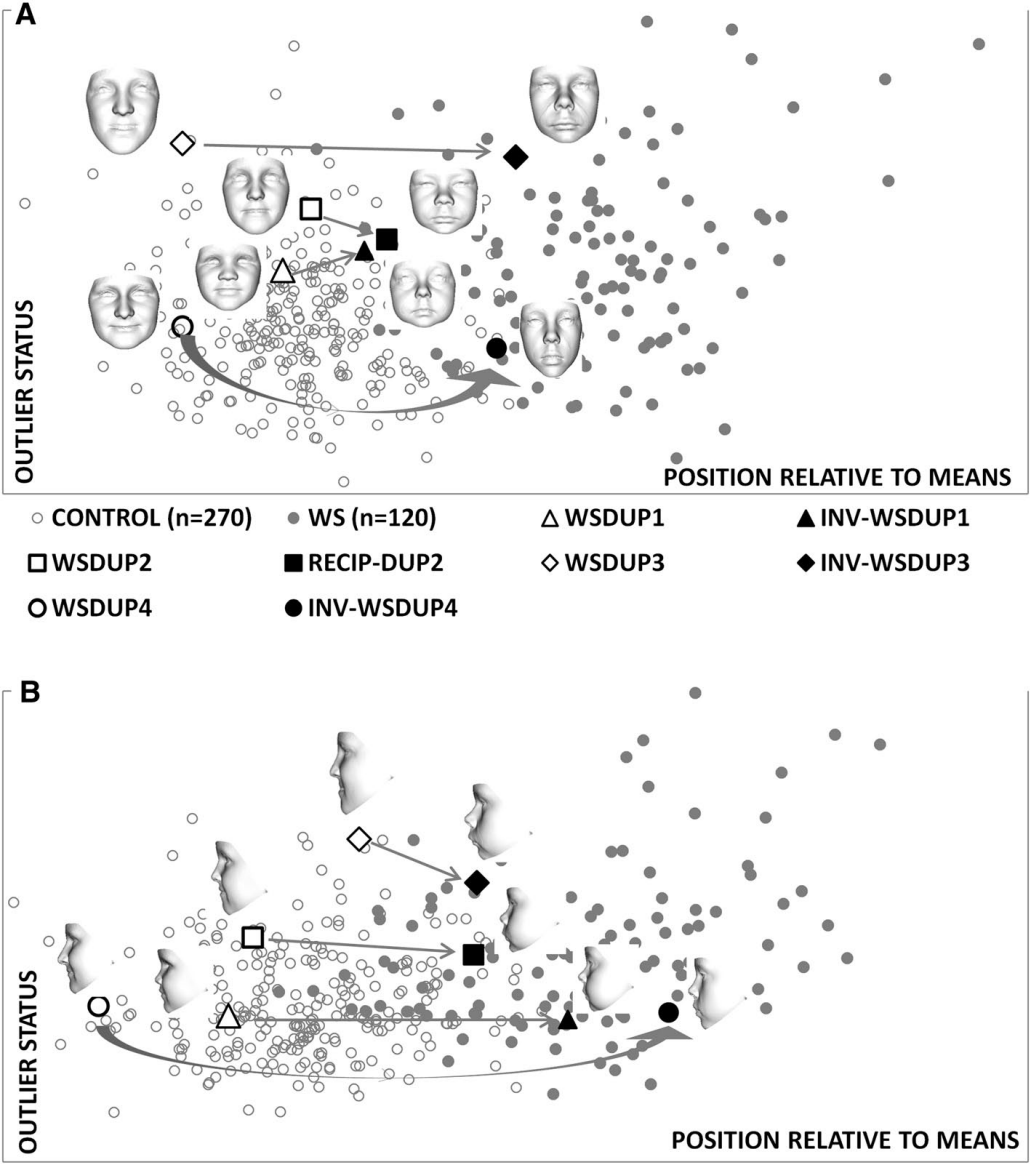
Closest mean classification of inversions of dup7q11.23 cases. The *arrows* emphasise position change in closest mean classification for the faces of the duplication cases in Fig. 1 **f**–**h** and their normative inversions. The horizontal axis determines relative similarity to the mean of the control group compared to the mean of the affected group. The vertical axis reflects the outlier status in terms of distance from the hyperline linking the means of the two groups under comparison. In **a**, the inversions of two duplication cases are classified at the periphery of the Williams–Beuren syndrome cluster. A third duplication inverts to well within the Williams–Beuren syndrome cluster. In **b**, when only the curvilinear mid-line facial profile is considered, all inversions are within the Williams–Beuren syndrome cluster. This is consistent with clinical evaluation suggesting the inverted faces to be somewhat wider than the typical Williams-Beuren syndrome facies but very Williams–Beuren syndrome-like in nose, lips and mid-line profile

We computed the change in relative similarity to the average faces of controls and individuals with Williams–Beuren syndrome resulting from normative inversion of the faces of each duplication 7q11.23 case. Using the full face without ears, the inverted facial forms are classified at the periphery of, or within, the Williams-Beuren syndrome cluster (Fig. 3a). Thus, normative inversion produces considerable position change from proximity to the average control towards the average Williams–Beuren syndrome face. For a thin ribbon-like surface along the mid-line facial profile (see Fig. S1 in supplementary material), normative inversion of all three duplication 7q11.23 cases results in even stronger classification within the Williams-Beuren syndrome cluster (Fig. 3b). These quantitative results confirm the clinical interpretation of the inverted face signatures as being Williams–Beuren syndrome-like, especially in the facial mid-line.

**Fig. 4:**
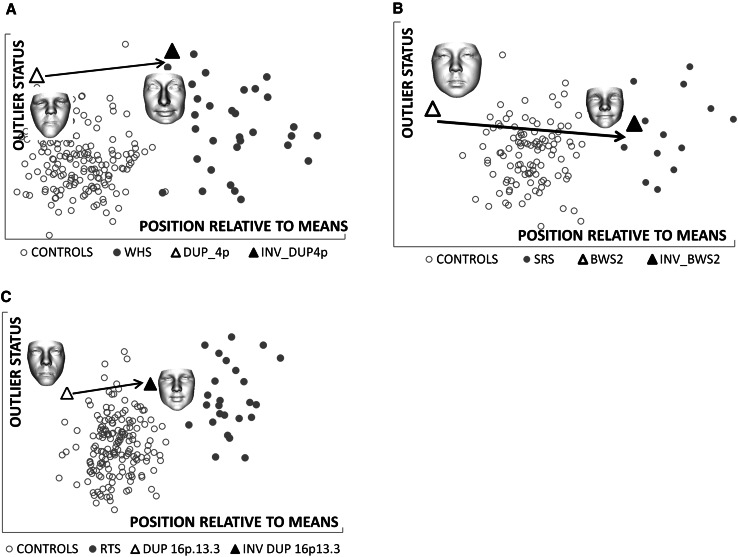
Closest mean classification of face for individuals with duplications of 4p16.3, 16p13.3 and H19 hypermethylation, and their normative inversions. Scatter plots **a**–**c** depict the results of closest mean classification of paired groups: controls and affected individuals with Wolf–Hirschhorn syndrome, Silver–Russell syndrome or Rubinstein–Taybi syndrome. The *arrows* indicate the change in closest mean classification position of an individual and their normative inversion. In each case, the result of the normative inversion is to alter the original face to be more like individuals in the affected group. Each *scatter* provides quantitative corroboration of the clinical evaluation of the face shape change resulting from the inversion

**Fig. 5:**
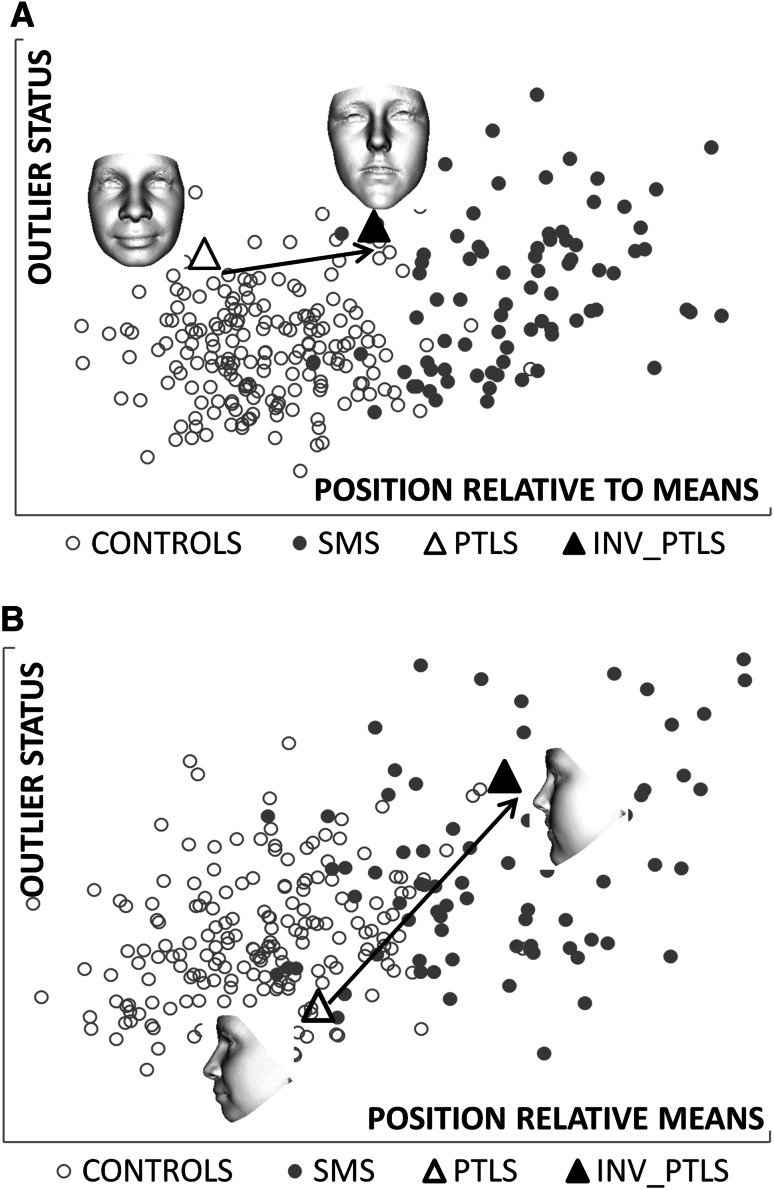
Closest mean classification of face and mid-line profile for individual with duplication of 17p11.2 and their normative inversions. *Scatter* plots **a** and **b** depict the result of closest mean classification of controls and individuals with Smith–Magenis syndrome. The *arrows* indicate the change in closest mean classification position of an individual and their normative inversion. In each case, the result of the normative inversion is to alter the original face or mid-line profile to be more like individuals in the affected group. Each scatter provides quantitative corroboration of the clinical evaluation of the shape change resulting from the inversion

Classification of face shape using the closest mean algorithm also produced positive recognition of the relevant facial characteristics for the other four genomic regions (Figs. 4, 5). For the Smith–Magenis and Potocki–Lupski syndromes comparison at 17p11.2, an additional shape classification for the mid-line facial profile was undertaken to demonstrate once again that shape inversion of a more localised region can produce more convincing objective results (Fig. 5).

The paucity of cases with duplications meant that we could not even carry out a compromise control-duplication axial comparison to provide more objective evidence of the effect of normative facial inversion on deletion cases. Instead, we applied normative inversion to individuals with Wolf–Hirschhorn, Williams–Beuren, Silver–Russell, Rubinstein–Taybi and Smith–Magenis syndromes to demonstrate the similarity of their inversions to individuals in the published literature with corresponding duplications/mutations at 4p16.3, 7q11.23, 11p15, 16p13.3 and 17p11.2 (supplementary Figs. S2–S6). In each of these supplementary figures, normative inversion is the only process that has been applied and only to the faces of the individuals with Wolf–Hirschhorn, Williams–Beuren, Silver–Russell, Rubinstein–Taybi and Smith–Magenis syndromes. To demonstrate the general effect of normative inversion, we also generated animated morphs between an average syndromic face and its normative inversion for each of Rubinstein-Taybi, Silver-Russell, Smith–Magenis, Williams–Beuren and Wolf–Hirschhorn syndromes (Supplementary videos SV1–SV5).

**Fig. 6:**
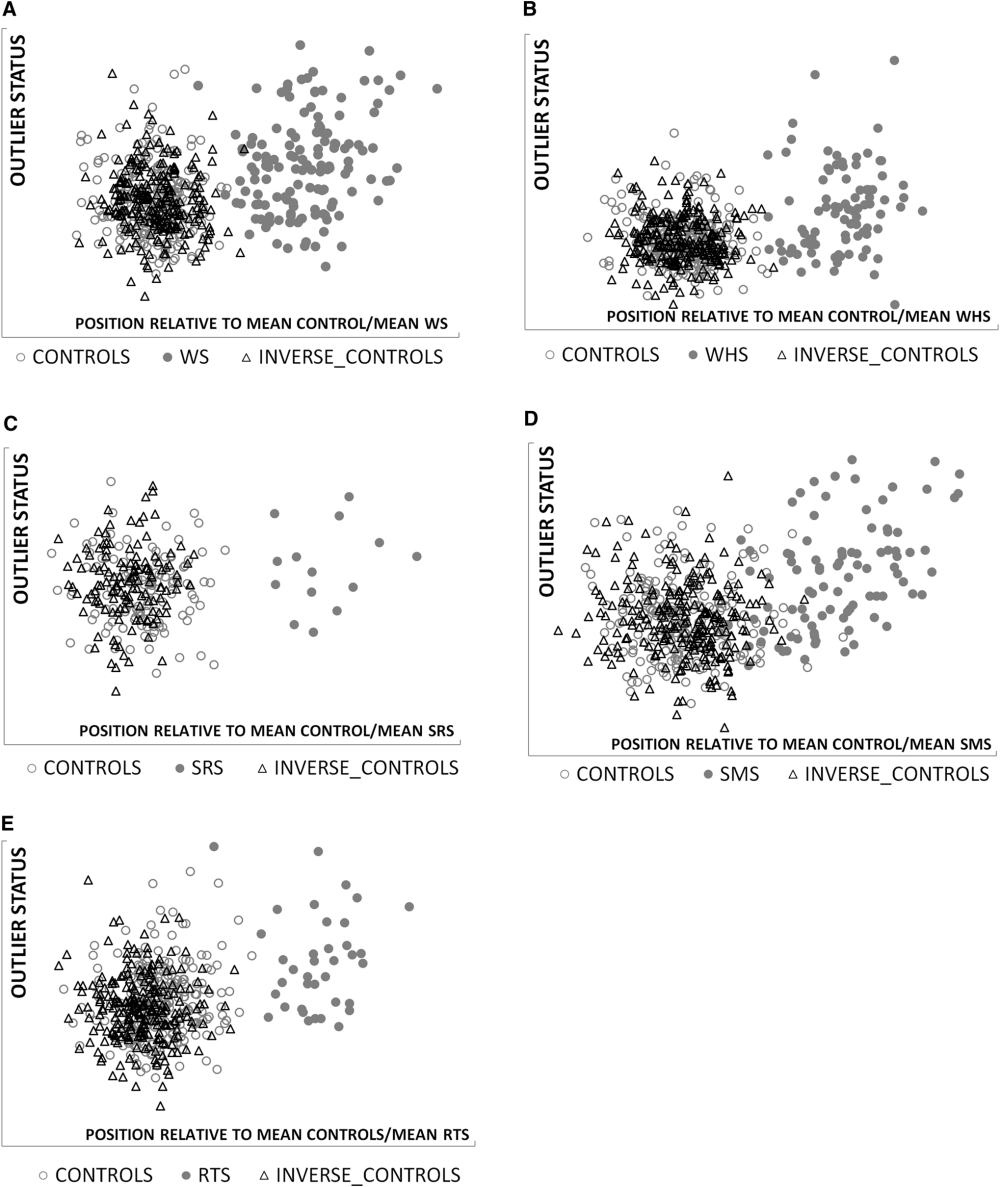
Closest mean classification of inverted controls against original controls and affected subgroups. Each *scatter* shows the closest mean classification of the normative inversion of controls with respect to the means of the original control and syndrome subgroup. In each case, the inverted controls cluster with the original controls and do not show evidence of facial features of the syndrome. **a**: Williams–Beuren syndrome **b**: Wolf–Hirschhorn syndrome **c**: Silver–Russell syndrome **d**: Smith–Magenis syndrome **e**: Rubinstein–Taybi syndrome

In order to check the effect of normative inversion on unaffected controls, we used multi-folded cross validation, employing closest mean classification to determine discriminating differences between 387 controls and their inverted forms. The average discrimination rate of 20 %, much lower than even chance classification, demonstrates that as a group normative inversions of control faces are indistinguishable from originals i.e., they fall within typical facial growth and development. Finally, to detect any possibility of facial inversion producing features similar to those of Williams–Beuren syndrome, we also tested closest mean classification of normatively inverted controls in a control-Williams–Beuren syndrome combined DSM. The resulting classification clearly demonstrates that inversion does not introduce Williams–Beuren-like facial characteristics (Fig. 6a). This was repeated for Wolf–Hirschhorn, Silver–Russell, Rubinstein–Taybi and Smith–Magenis syndromes, all with similar negative results (Fig. 6b–e).

## Conclusion

We have demonstrated the efficacy of normative inversion of facial form in the investigation of gene-dosage effects at 4p16.3, 7q11.23, 11p15, 16p13.3 and 17p11.2. There is a resemblance between normatively inverted faces of individuals with a duplication or (epi-)mutation and the facial characteristics of deletion or mutation cases, and vice versa. On chromosome 4p16.3, the genes *TACC3*, *FGFR3*, and *LETM1* have been shown to be dosage sensitive (Cyr et al. 2011). Two related genes on 7q11.23, *GTF2IRD1* and *GTF2I*, have been implicated in the cause of craniofacial dysmorphism in Williams–Beuren syndrome and there is evidence of dosage-sensitivity (Tassabehji et al. 2006). BAZ1B has also been implicated in craniofacial development in Williams–Beuren syndrome (Ashe et al. 2008), although its role is still unclear (Yoshimura et al. 2009). Dosage sensitivity of *CREBBP* was established by the identification of low-level mosaic individuals with a typical Rubinstein–Taybi syndrome phenotype (Gervasini et al. 2007). Mouse studies of *RAI1* have also identified opposite behavioural phenotypes with respect to either deletion or duplication (Carmona-Mora and Waltz 2010). Here, we have demonstrated that face shape inversion can be used to investigate how diametric changes in gene dosage influence craniofacial form.

In studying duplication cases, clinicians often remain uncertain about their pathogenicity, and it is often difficult to determine reliably whether or not the facial dysmorphism in a patient is consistent. It might be useful, therefore, to compare the inverted face of an individual with a duplication of uncertain significance to faces of individuals with deletions or mutations in genes of the same region in whom the phenotype has been more clearly defined. This approach would, for example, be useful for screening individuals with uncertain CNVs recorded in on-line databases such as Decipher (Firth et al. 2009). Conversely, the inverted faces of individuals with a deletion or a mutation could be a useful visualisation of possible facial features associated with duplications of associated genomic regions, especially those containing known dosage sensitive genes, which should assist recognition.

The inverted facial form we have prescribed is simply defined but by the same token is a rather gross transformation to apply across the entire face. More localised application, for example to facial profile or perinasal and periorbital regions, will sometimes be more appropriate. Larger numbers of age, sex and ethnicity matched controls will improve the accuracy of matched norms and normative inversions. Animal studies, such as the recent linkage of non-coding regions to facial form (Attanasio et al. 2013), using normative transformation of facial and cranial structures will be an appropriate route for determining where, to what degree, and at what stage, specific genes produce dosage-sensitive effects on facial, cranial and potentially brain development (Crespi 2013). Our results have taken an initial step in demonstrating the use of normative inversion of human faces in the study of gene dosage sensitivity.

## Electronic supplementary material

Below is the link to the electronic supplementary material.

One Microsoft WORD file containing six figures (SUPPLEMENTARY_MATERIAL_Hammond_FEB_18th_2014.docx).

One zipped file of separate PDF files describing permission rights for the supplementary figures previously published elsewhere.

Five animated morphs from an average syndromic face to its normative inversion for each of Rubinstein-Taybi, Silver-Russell, Smith-Magenis, Williams-Beuren and Wolf-Hirschhorn syndromes.

Supplementary material 1 (DOCX 4843 kb)

Supplementary material 2 (AVI 637 kb)

Supplementary material 3 (AVI 752 kb)

Supplementary material 4 (AVI 589 kb)

Supplementary material 5 (AVI 658 kb)

Supplementary material 6 (AVI 735 kb)
