# Supplementary material for: Opposite effects on facial morphology due to gene dosage sensitivity
Source: Hum Genet. 2014 Jun 3;133(9):1117–25. doi: 10.1007/s00439-014-1455-z (PMC4148161; doi:10.1007/s00439-014-1455-z)
Supplement: Supplementary file 1 — Supplementary material 1 (DOCX 4843 kb) [file 439_2014_1455_MOESM1_ESM.docx]

**Figure S1**

**
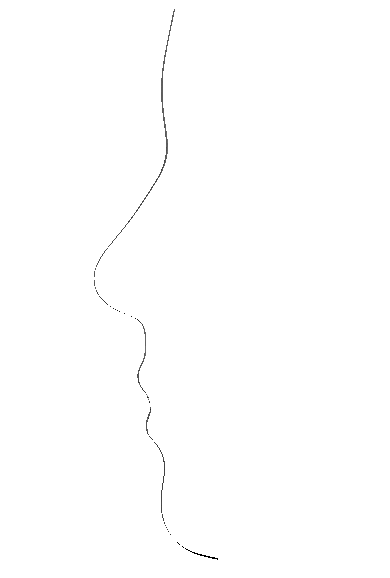

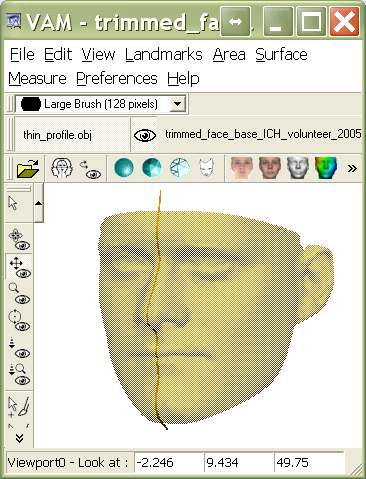
**

The thin ribbon like surface used to build dense surface models of the mid-line profile of the face.

**Figure S2**


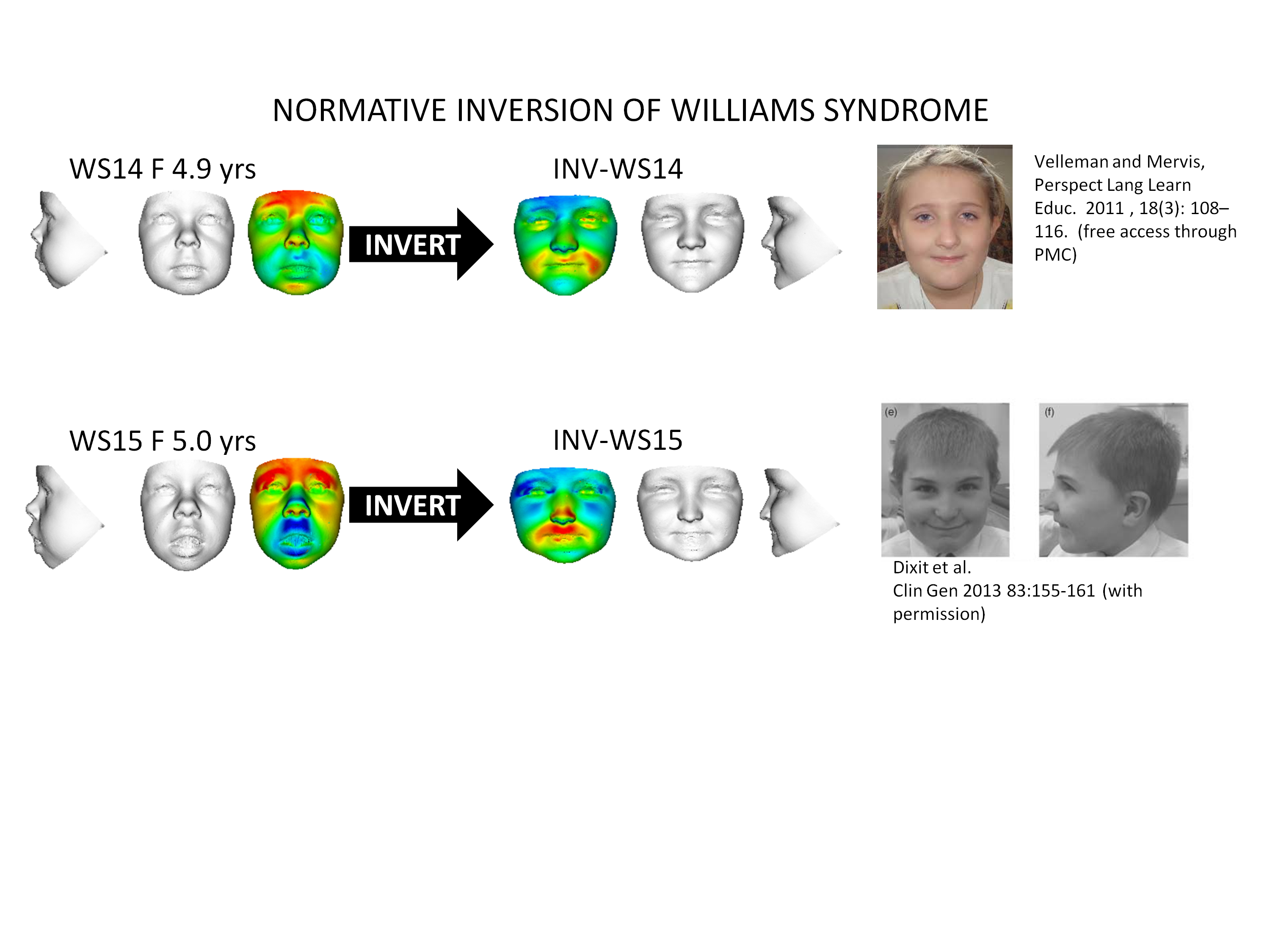
Two examples are shown of individuals with a confirmed deletion of 7q11.23. Each row contains a triptych of profile, portrait and signature of the individual; a similar triptych for the normative inversion of the individual; and an example of a case of a duplication of 7q11.23 taken from the published literature to demonstrate similarity with the inversion of the deletion case.

**Figure S3**


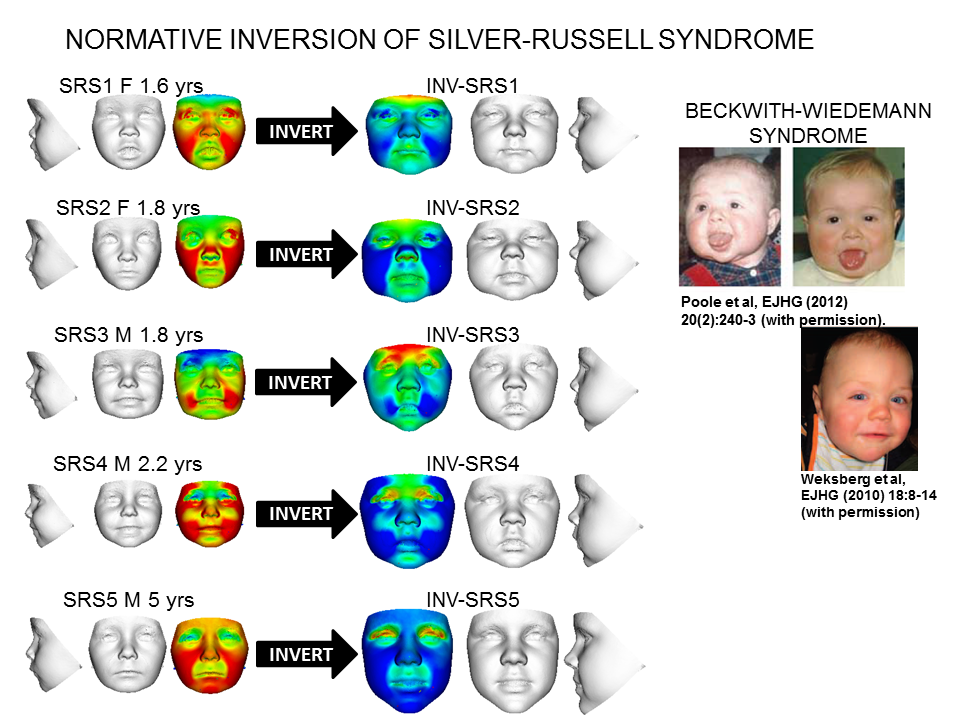


Five examples are shown of individuals with a confirmed diagnosis of Silver-Russell syndrome caused by hypomethylation of H19. Each row contains a triptych of profile, portrait and signature of the individual; and a similar triptych for the normative inversion of the individual. A few examples are provided of published cases of Beckwith-Wiedemann syndrome caused by hypermethylation of H19 to demonstrate similarity with the inversion of the inverted Silver-Russell syndrome cases. In particular, the inverted faces have short, bulbous noses, a broad zygomatic arch with full cheeks emphasising the point of the chin.

**Figure S4**


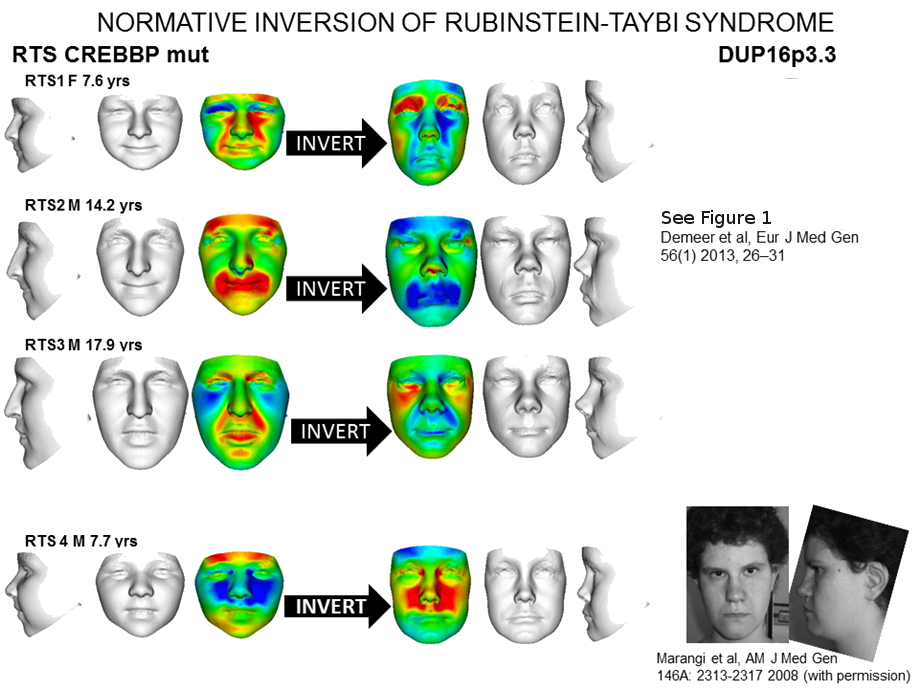


Four examples are shown of individuals with a confirmed diagnosis of Rubinstein-Taybi syndrome due to a *CREBBP* mutation at 16p13.3. Each row contains a triptych of profile, portrait and signature of the individual; a similar triptych for the normative inversion of the individual; and an example of a case of a duplication of 16p13.3 taken from the published literature to demonstrate similarity with the inversion of the deletion case. Notice, in particular, that the inverted faces have a short upturned nose due to the concave nasal ridge; malar flattening and a similar zygomatic and gonial width. In contrast, the opposite is true of the Rubinstein-Taybi syndrome face with a convex nasal ridge, anteriorly protruding zygoma and very broad mid-face.

**Figure S5**


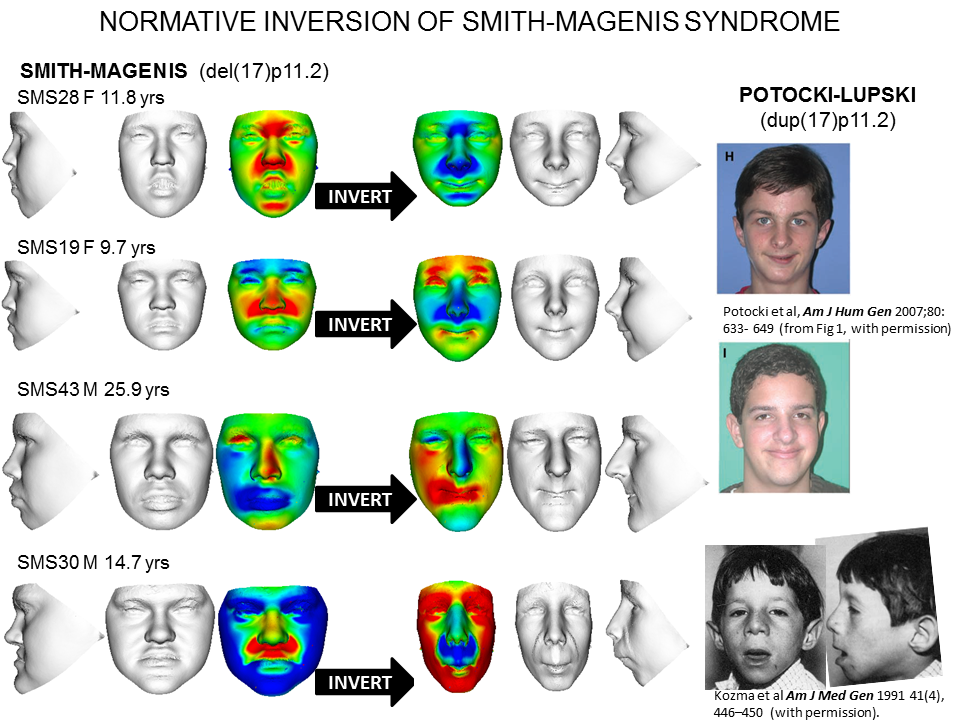


Four examples are shown of individuals with a confirmed diagnosis of Smith-Magenis syndrome due to a deletion of 17p11.2. Each row contains a triptych of profile, portrait and signature of the individual; a similar triptych for the normative inversion of the individual; and an example of a case of Potocki-Lupski syndrome caused by a duplication of 17p11.2 taken from the published literature to demonstrate similarity with the inversion of the Smith-Magenis syndrome case. Notice, in particular, in Smith-Magenis syndrome the nose shape is broad at the tip with a hidden columella as if the subnasale has been pushed upwards, and to some extent posteriorly, producing mid-facial hypoplasia. The inverted Smith-Magenis syndrome faces necessarily have a narrower nasal tip, a more obvious columella and mid-facial prominence. These are features seen in the Potocki-Lupski syndrome cases. Another interesting feature is the appearance of downturned corners of the mouth in the inverted Smith-Magenis syndrome cases. This is a feature commented on in the literature on Potocki-Lupski syndrome. The final Smith-Magenis syndrome example shows extreme mid-facial hypoplasia which when inverted necessarily becomes more anteriorly prominent and similar to the final published case shown.

**Figure S6**


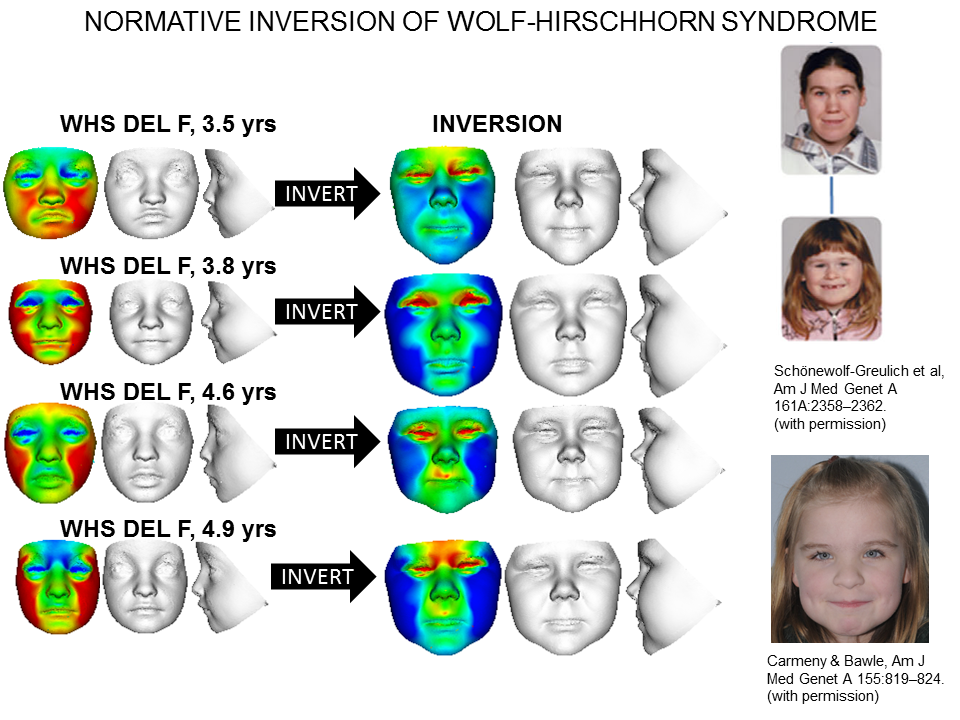


Four examples are shown of individuals with a confirmed diagnosis of Wolf-Hirschhorn syndrome due to a terminal deletion of 4p. Each row contains a triptych of profile, portrait and signature of the individual. A few examples of duplication 4p cases from the published literature are included to demonstrate similarity with the inversion of the Wolf-Hirschhorn syndrome cases. Notably, the open, upsweep to the Wolf-Hirschhorn syndrome orbit and supra-orbit inverts to one that is shorter and more deeply set. The relatively long nose with a tendency to an outward curve and flat tip inverts to one that is shorter and more bulbous at the tip. The much reduced gonial width in Wolf-Hirschhorn syndrome inverts to a very square jaw. The example duplication cases from the published literature show many of the features of the Wolf-Hirschhorn syndrome inversions.
